# Acute multifocal retinitis in a patient with Q fever (Coxiella Burnetii infection) with endocarditis

**DOI:** 10.1186/s12348-022-00295-1

**Published:** 2022-06-20

**Authors:** Anis Mahmoud, Fatma Abid, Molka Khairallah, Sofien Affes, Sameh Mbarek, Hassen Ibn Hadj Amor, Anis Ben Hadj Khalifa, Riadh Mesaoud, Moncef Khairallah

**Affiliations:** 1 Department of Ophthalmology, Tahar Sfar University Hospital, Mahdia, Tunisia; 2 Department of Cardiology, Tahar Sfar University Hospital, Mahdia, Tunisia; 3 Department of Bacteriology, Tahar Sfar University Hospital, Mahdia, Tunisia; 4 Department of Ophthalmology, Fattouma Bourguiba University Hospital, Monastir, Tunisia

**Keywords:** Acute multifocal retinitis, Acute Q fever, Coxiella burnetii, endocarditis

## Abstract

**Objective:**

To report acute multifocal retinitis in association with serologically-proven *Coxiella (C) Burnetii* infection (Q fever) with endocarditis.

**Material and methods:**

A single case report documented with multimodal imaging.

**Results:**

A 67-year-old cattle breeder presented with a 2-week history of persistent fever, headache, and floaters in both eyes. On examination, his best-corrected visual acuity was 20/20, and there was 1+ vitreous cells in both eyes. Bilateral fundus examination showed multiple small superficial white retinal lesions scattered in the posterior pole and midperiphery associated with a few retinal hemorrhages. These retinal lesions did not stain on fluorescein angiography (FA) and showed focal hyperreflectivity and thickening primarily involving the inner retinal layers on optical coherence tomography (OCT). There also was a band-like hyper-reflective area in the middle retina consistent with paramacular acute middle maculopathy. Transthoracic echocardiogram (TTE) showed a mobile echodensity on the anterior aortic leaflet consistent with a diagnosis of endocarditis. Elisa assays performed on paired serum samples collected 2 weeks apart showed increase in *antibodies* against *C* burnetii from 60 IU/ml to 255 IU/ml. The patient was treated with doxycycline 100 mg twice a day for 18 months, with subsequent resolution of the endocarditis. Sequential ocular examinations showed gradual resolution of all acute retinal findings without visible scars.

**Conclusion:**

Acute Q fever, caused by *C burnetii* infection, should be considered in the differential diagnosis of acute multifocal retinitis. A systematic cardiac assessment with echocardiography is essential for early diagnosis of associated endocarditis and for prompt administration of appropriate antibiotic treatment to improve clinical outcomes**.**

## Introduction

Q fever is a worldwide distributed zoonosis caused by *C burnetii,* an obligate gram-negative intracellular organism [1]. It is primarily transmitted to humans through inhalation of aerosols from contaminated soil or animal waste, with cattle, sheep, and goats being the main reservoirs. Incubation period ranges from 2 to 50 days with a median of 18 days [2]. Most patients remain asymptomatic or develop a nonspecific and self-limiting febrile illness, so that Q fever remains frequently undiagnosed. Endocarditis is the most common and most serious manifestation of chronic Q fever, but other severe acute or chronic complications also have been described including pneumonia, hepatitis, osteomyelitis, endovascular infection, and involvement of the central nervous system [3, 4].

Ocular involvement has rarely been described in the course of Q fever including anterior and posterior uveitis, optic neuropathy, exudative retinal detachment, and abducens palsy [5]. We herein describe a patient who developed acute multifocal retinitis (AMR) in association with acute *C burnetii* infection with endocarditis.

## Case report

**Fig. 1:**
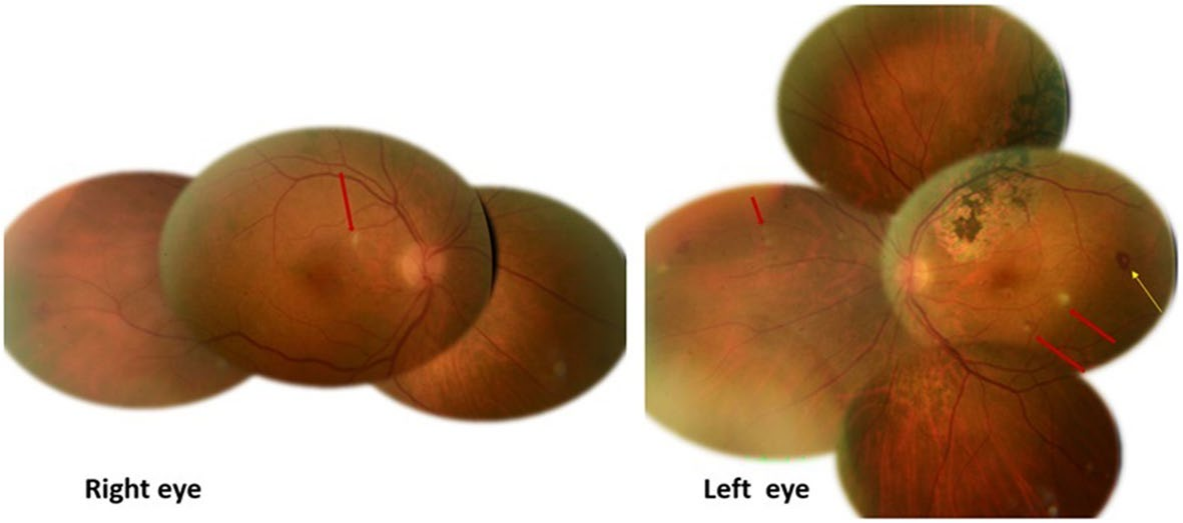
Baseline composite fundus photography shows bilateral small yellow-white retinal lesions in the posterior pole and the periphery (red arrows), with a few retinal hemorrhages, some of which are white-centered (yellow arrow). Note the presence of an old, flat, well-delineated atrophic and pigmented lesion along the superotemporal retinal vascular arcade in the left eye

A 67-year-old cattle breeder presented to the emergency department with a 2-week history of persistent fever, headache, and floaters in both eyes. On examination, his best-corrected visual acuity was 20/20 in both eyes. There was no relative afferent pupillary defect, and the ocular motility examination was normal for both eyes. Slit-lamp examination showed a quiet anterior chamber and 1+ vitreous cells bilaterally. Intraocular pressure was 12 mmHg in both eyes. Fundus examination revealed multiple white spots at the level of the inner retina, measuring 200 to 500 microns, scattered in the posterior pole and midperiphery in both eyes. There also were a few retinal hemorrhages, with some them having a white center. A flat, well-circumscribed chorioretinal lesion associating atrophic and hyperpigmented areas was seen along the superotemporal vascular arcade in the left eye (LE) (Fig. 1).

FA showed a masking effect from retinal hemorrhages, a slight hypofluorescence of retinal infiltrates without late staining, and peripheral retinal vascular leakage.

**Fig. 2:**
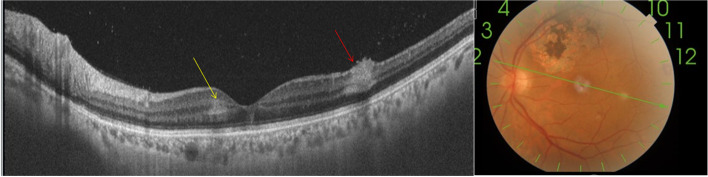
Swept source OCT scan of the LE passing through a retinal infiltrate shows hyperreflective preretinal vitreous dots and a focal area of thickened, hyperreflective inner retina with infiltration extending from the nerve fiber layer to the outer retinal layers, with the ellipsoid zone, retinal pigment epithelium, and choroid clearly delineated and spared (red arrow). Note the presence of a band-like hyperreflective area in the middle retina suggestive of PAMM (yellow arrow)

Swept source-OCT scan (Topcon, DRI triton) through a retinal infiltrate in the LE revealed a focal area of retinal hyperreflective thickening extending from the retinal nerve fiber layer to the outer retinal layers, with sparing of the retinal pigment epithelium and choroid. There also was a band-like hyper-reflective area in the middle retina consistent with paramacular acute middle maculopathy (PAMM) (Fig. 2).

**Fig. 3:**
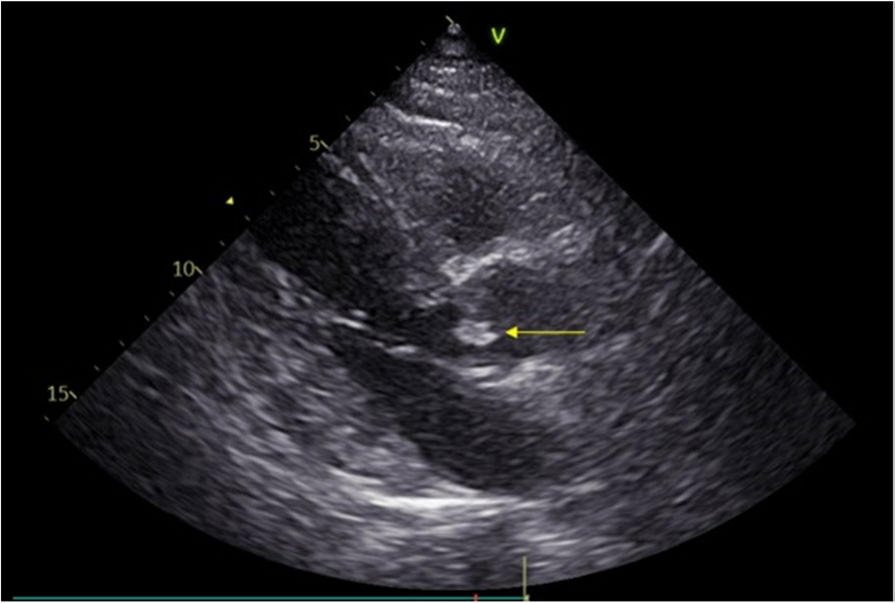
Transthoracic echocardiogram shows a mobile echodensity on the anterior aortic leaflet measuring 20 x 14 mm consistent with endocarditis (yellow arrow)

Cardiac examination showed a diastolic decrescendo murmur. TTE showed a mobile echodensity on the anterior aortic leaflet measuring 20 x 14 mm consistent with a diagnosis of endocarditis (Fig. 3).

Repeated blood cultures were negative. A lumbar puncture was performed revealing an aseptic meningitis with increased lymphocyte and protein levels. Laboratory testing was negative for bartonellosis, rickettsial infection, syphilis, and tuberculosis.

The diagnosis of acute Q fever was made on the basis of SERION enzyme-linked immunosorbent assay (ELISA) results with positive *C burnetii* phase II IgM and increase in *IgG titers* from 60 IU/ml to 255 IU/ml on paired serum samples collected 2 weeks apart. The patient was treated with doxycycline 100 mg twice a day for 18 months.

**Fig. 4:**
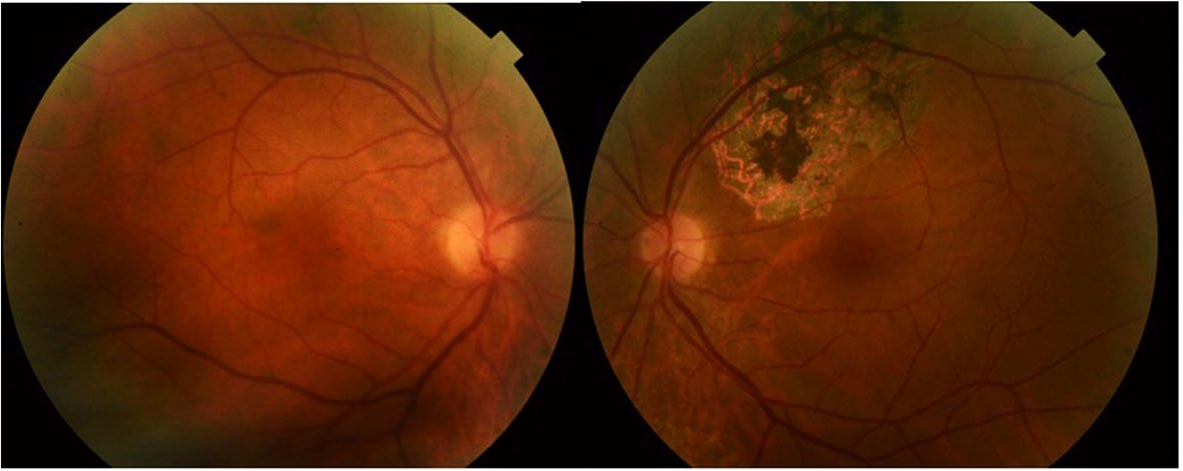
Fundus photography taken six weeks after initial presentation shows a complete resolution of retinal hemorrhages and multifocal retinal lesions, without visible scarring

**Fig. 5:**
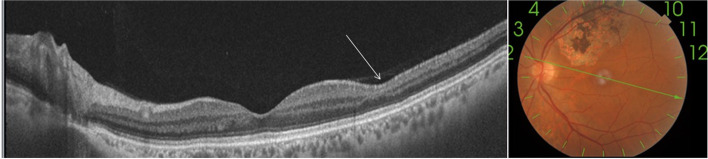
Swept source-OCT scan taken at the same time shows the disappearance of the band-like hyper-reflectivity corresponding to PAMM and a focal area of inner retinal thinning corresponding to a resolved retinal infiltrate (arrow)

Six weeks after initial presentation, the patient reported the disappearance of floaters. Fundus examination showed a complete resolution of retinal hemorrhages and multifocal retinal lesions, with no residual chorioretinal scars (Fig. 4). Swept source-OCT scan showed the disappearance of the band-like hyper-reflective area in the middle retina corresponding to PAMM and a focal area of inner retinal thinning corresponding to a resolved retinal infiltrate (Fig. 5). Sequential TTE over a two-month follow-up period showed gradual regression of the endocardial vegetation.

## Discussion

Our report is the first to describe the association of AMR with a serologically proven systemic *C burnetii* infection. AMR, previously termed acute multifocal inner retinitis, has been considered to be often idiopathic, but several infectious etiologies have been recognized including cat scratch disease, rickettsial infection, and syphilis [6–9]. Recent data from Tunisia show rickettsial disease, including rickettsia conorii and rickettsia typhi, to be the most common cause of AMR [10]. In the present case, serological testing showed negative results for rickettsial infection and also for cat scratch disease and syphilis. The presence of an associated blood-culture negative endocarditis in a patient with a history of animal contact raised our suspicion of Q fever. The diagnosis was confirmed by the detection of high titers of anti- *C burnetii antibodies* and the patient accordingly was treated with oral doxycycline. Indirect immunofluorescent assays (IFA) is considered the gold standard for the diagnosis of Q fever ; alternatively, ELISA may be done [11].

The ocular disease pattern of our patient conformed to most of the previously reported features of AMR, especially with regard to the systemic febrile illness, minimal vision impairment, characteristic multiple inner retinitis spots associated with mild vitritis, and benign and self-limiting clinical course.

Endocarditis has been considered as an almost exclusive complication of chronic Q fever. Our findings, consistent with previous recent data, show that acute endocarditis, readily detectable with TTE, can affect a sizable subset of patients with acute Q fever [12–14]. Therefore, early diagnosis of Q fever and associated endocardial involvement is of utmost importance for prompt initiation of antibiotic treatment to prevent persistent endocarditis and related morbidity and mortality.

Multifocal superficial retinal infiltrates associated with Q fever may result from intraretinal multiplication of *C burnetii*, or from immune-mediated response to bacterial antigens caused by the deposition of immune complexes, inflammatory cells, or antibodies through retinal vessels [15, 16]. The vascular tropism of *C burnetii* also is reflected in our patient in the presence of associated retinal hemorrhages, retinal vascular leakage, and retinal vascular occlusion in the form of PAMM.

This case shows that *C burnetii* infection should be considered in the differential diagnosis of AMR associated with systemic febrile illness. Serological confirmation is required for a definitive diagnosis that could be challenging. A systematic echocardiography is of utmost importance for early detection of disease-related endocarditis and for prompt administration of appropriate antibiotic treatment to improve clinical outcomes.

## Data Availability

The datasets used and/or analysed during the current study are available from the corresponding author on reasonable request.

## Abbreviations

AMR: Acute Multifocal Retinitis
C: Coxiella
FA: Fluorescein Angiography
LE: Left Eye
OCT: Optical Coherence Tomography
PAMM: Paramacular Acute Middle Maculopathy
TTE: Transthoracic Echocardiogram
